# Non-Contact Adaptive Voltage Sensor Based on Electric Field Coupling Principle

**DOI:** 10.3390/s23198316

**Published:** 2023-10-08

**Authors:** Xiangyu Tan, Wenbin Zhang, Mingxing He, Wenyun Li, Gang Ao, Fangrong Zhou

**Affiliations:** 1 Electric Power Research Institute, Yunnan Power Grid Co., Ltd., Kunming 650217, China; 2 College of Mechanical and Electrical Engineering, Kunming University of Science and Technology, Kunming 650504, China; 3 College of Science, Kunming University of Science and Technology, Kunming 650504, China; 4 China Southern Power Grid, Yunnan Power Grid Co., Ltd., Kunming 650011, China; 5 Yunnan Power Grid Co., Ltd., Kunming Power Supply Bureau, Kunming 650001, China

**Keywords:** electric field coupling, non-contact voltage measurement, variable voltage division ratio, self-adaption

## Abstract

Non-contact voltage sensors based on the principle of electric field coupling have the advantages of simple loading and unloading, high construction safety, and the fact that they are not affected by line insulation. They can accurately measure line voltage without the need to connect to the measured object. Starting from the principle of non-contact voltage measurement, this article abstracts a non-contact voltage measurement model into the principle of capacitive voltage sharing and deduces its transfer relationship. Secondly, it is theoretically inferred that the edge effect of the traditional symmetric structure sensor plate will cause the actual capacitance value between the sensor plates to be greater than the theoretically calculated capacitance value, resulting in a certain measurement error. Therefore, the addition of an equipotential ring structure is proposed to eliminate the edge additional capacitance caused by the edge effect in order to design the sensor structure. In addition, due to the influence of sensor volume, material dielectric constant, and other factors, the capacitance value of the sensor itself is only at pF level, resulting in poor low-frequency performance and imbuing the sensor with a low voltage division ratio. In this regard, this article analyzes the measurement principle of non-contact voltage sensors. By paralleling ceramic capacitors between the two electrode plates of the sensor, the capacitance of the sensor itself is effectively increased, improving the low-frequency performance of the sensor while also increasing the sensor’s voltage division ratio. In addition, by introducing a single pole double throw switch to switch parallel capacitors with different capacitance values, the sensor can have different voltage division ratios in different measurement scenarios, giving it a certain degree of adaptability. The final sensor prototype was made, and a high and low voltage experimental platform was built to test the sensor performance. The experimental results showed that the sensor has good linearity and high measurement accuracy, with a ratio error of within ±3%.

## 1. Introduction

In the power system, line voltage, as a basic data point, plays a very important role in numerous engineering projects and theoretical research [1,2,3,4]. Voltage measurement, as an important component of the power system, plays a decisive role in applications such as relay protection [5], energy metering, intelligent equipment control [6,7], and the online monitoring of overvoltage [8,9,10,11]. The accuracy, reliability, convenience, and speed of voltage measurement are key technical requirements in energy metering and relay protection, power system monitoring and diagnosis, and power system fault analysis, and they are the foundation for ensuring the safe operation of the power system. In the current power system, voltage signals are mainly obtained through voltage transformers. The most commonly used voltage transformers in the current power system are electromagnetic voltage transformers and capacitive voltage transformers. However, with the gradual improvement in the voltage level of the power grid, in some aspects, the defects of the traditional form of voltage transformers are gradually exposed and cannot meet the development needs of the current power system [12,13,14,15,16]. With the increasing pace of smart grid construction, the power system is rapidly developing towards the goals of intelligence, digitization, and automation. In addition to stability and reliability, voltage measurement will also face the needs and challenges of being smaller, digital, and more convenient [17]. However, traditional voltage measurement methods are no longer able to meet the current development needs of power systems. Therefore, there is an urgent need for a low-cost, widely applicable, and structurally simple voltage measurement device for voltage measurement. The non-contact voltage sensor based on the principle of electric field coupling facilitates convenient measurements and has the advantages of a simple structure, a wide linear range of measurement, a stable transient response, and a fast response speed. It has facilitated a new method of voltage measurement to meet the measurement requirements of the current power system [18,19,20,21,22]. Non-contact voltage measurement has been widely studied and applied in power measurement, the online monitoring of overvoltage, partial discharge monitoring, and equipment automation [23,24].

The non-contact voltage sensor based on the principle of electric field coupling forms a coupling capacitance (high-voltage capacitance) between the sensing electrode and the line to be tested and forms its own mutual capacitance (low-voltage capacitance) between the two sensing electrodes of the sensor. The voltage of the line to be tested is measured by the voltage attenuation of the high-voltage capacitance and the low-voltage capacitance in series. The authors of [25,26,27] conducted relevant research on non-contact voltage sensors based on the principle of electric field coupling. Traditional voltage sensors cannot be used together with integrators or attenuators in actual measurement scenarios due to their own transfer function, making them applicable to measuring high voltage signals at specific high frequencies and resulting in their measurement frequency band being small. The size of high-voltage and low-voltage capacitors directly determines the low-frequency performance and partial voltage ratio of voltage sensors. In order for the sensor to have good low-frequency performance and a large partial voltage ratio, the mutual capacitance between the two sensing electrodes of the sensor, namely the low-voltage capacitance, should be large enough. However, often influenced by factors such as the size of the sensor volume and the dielectric constant of the insulation medium between the electrode plates, low-voltage capacitors cannot have large capacitance values, resulting in poor low-frequency performance of the sensor, a small partial voltage ratio, and difficulty in making adjustments. In addition, due to the edge effect of the symmetric structure sensor plate, there is an additional capacitance at the edge of the sensor plate, resulting in the actual capacitance value of the sensor being greater than the theoretically calculated capacitance value.

For this paper, by designing the structure of the sensing electrode, we proposed the notion of adding an equipotential ring to the electrode structure to eliminate the edge effect between the plates so that the true capacitance value of the sensor is equal to the theoretically calculated capacitance value. By paralleling ceramic capacitors between the two sensing electrodes of the sensor, the capacitance value of the sensor itself (low-voltage capacitance) can be effectively increased, making the sensor operate in self-integration mode to improve its low-frequency performance. The parallel capacitance value (nF level or even uF level) is often several orders of magnitude larger than the sensor’s own capacitance (pF level), which effectively improves the sensor’s partial voltage ratio and provides a foundation for measurement in high-voltage application scenarios. In addition, a single pole double throw switch was introduced to switch ceramic capacitors with different capacitance values. By changing the size of the parallel ceramic capacitors, the sensor has different partial voltage ratios to adapt to different high- and low-voltage measurement scenarios. The sensor has the advantages of being inexpensive, small in terms of sensor volume, and able to facilitate convenient adaptive adjustments to the partial pressure ratio, and the feasibility of this sensor has been ultimately verified through experiments.

## 2. Sensor Measuring Principle

### 2.1. Principles of Electric Field Coupling Measurement

**Figure 1:**
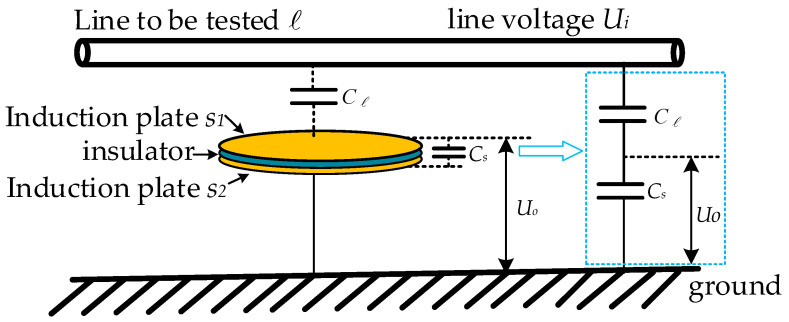
A non-contact voltage measurement model based on the principle of electric field coupling.

The non-contact voltage measurement model based on the principle of electric field coupling is shown in Figure 1. The upper and lower induction electrodes $S_{1}$ and $S_{2}$ of the voltage sensor and the insulating medium in the middle form the sensor‘s own capacitance (low-voltage capacitance) $C_{s}$, the upper induction electrode and the line to be tested form a coupling capacitance (high-voltage capacitance) $C_{l}$, and the lower induction electrode is connected to the earth to determine the reference potential of the output voltage. Equivalent to the traditional capacitor voltage division principle, the capacitor $C_{l}$ and the capacitor $C_{s}$ are connected in series to form a voltage divider. The line voltage $U_{i}$ to be tested is divided by $C_{l}$ and $C_{s}$, and the voltage $U_{o}$ is output from the capacitor $C_{s}$.

**Figure 2:**
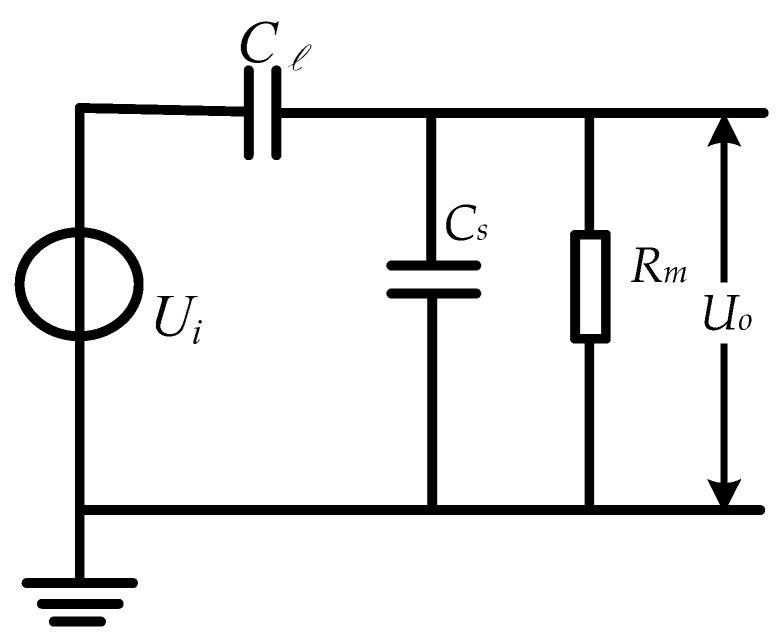
Equivalent circuit model for non-contact voltage measurement with lumped parameters.

Figure 2 shows the equivalent circuit model of the voltage measurement model in Figure 1. Here, $U_{i}$ corresponds to the voltage value of the tested line, $C_{l}$ is the coupling capacitance formed by the upper induction electrode and the tested line, $C_{s}$ is the mutual capacitance formed by the two induction electrodes and the intermediate insulation medium of the voltage sensor, and $R_{m}$ is the grounding load resistance.

The sensor can be equivalent to a first-order RC circuit composed of $C_{l}$, $C_{s}$, and $R_{m}$. Through Laplace transform, the transfer function of the sensor can be obtained as follows:

(1) $$H(s)=\frac{U_{o}(s)}{U_{i}(s)}=\frac{C_{l}R_{m}s}{(C_{l}+C_{s})R_{m}s+1}$$

when $(C_{l}+C_{s})R_{m}s<<1$, the transfer function can be expressed as follows:

(2) $$H(s)=\frac{U_{o}}{U_{i}}=C_{l}R_{m}s$$

In this case, the sensor operates in differential mode and requires the integration circuit to be added to the subsequent circuit in order to achieve a linear relationship between the measured output signal and the measured signal.

When $(C_{l}+C_{s})R_{m}s>>1$, the transfer function can be expressed as follows:

(3) $$H(s)=\frac{U_{o}}{U_{i}}=\frac{C_{l}}{C_{l}+C_{s}}$$

At this point, the sensor operates in self-integration mode, with $U_{o}$ being proportional $U_{i}$. The sensor does not require an integrator to make its input and output linear. The voltage division ratio $k_{1}$ of the sensor is as follows:

(4) $$k_{1}=\frac{U_{i}}{U_{o}}=\frac{C_{l}+C_{s}}{C_{l}}$$

From the above equation, it can be seen that the voltage division ratio of the sensor is determined by the size of the capacitors $C_{l\;}$ and $C_{s}$. The limitations in the volume size of the sensor and the small difference in capacitance values between $C_{l}$ and $C_{s}$ will limit the improvement in the voltage division ratio of the sensor, making it difficult to achieve the direct digitization of its output.

From the above analysis, it can be concluded that if the sensor operates in differential mode, an integrating circuit needs to be added to the subsequent circuit to make the output voltage signal linearly related to the voltage signal to be measured. However, using an integrating circuit can bring a series of problems, including the following: (1) it creates difficulties in improving the signal-to-noise ratio of the sensor; (2) it results in transmission waveform distortion due to the spurious parameters of the integration circuit elements; and (3) it makes the analog integration circuit inaccurate due to the limitations in the temperature coefficients of the capacitors and resistors. Aiming at a series of problems in the integration circuit, making the sensor work in the self-integration mode will have greater advantages. However, in order for the sensor to operate in self-integration mode, as $C_{l\;}$ and $C_{s}$ are generally in the pF level (10^−12^), in order to meet the requirements of $(C_{l}+C_{s})R_{m}s>>1$ to operate the sensor in self-integration mode, it is necessary to use a load resistor Rm with a resistance value of GΩ level. This will have many problems in selecting resistance and matching impedance, which cannot meet practical needs. At the same time, due to the small difference in capacitance values between $C_{l\;}$ and $C_{s}$, the voltage division ratio of the sensor is limited, making it difficult to directly digitize its output.

### 2.2. Adaptive Principle of Voltage Sensors

In order to solve the practical situation mentioned above, it is difficult for the sensor to meet the self-integration condition and work in the self-integration mode. Moreover, due to the small difference in capacitance values between $C_{l\;}$ and $C_{s}$, the voltage division ratio of the sensor is not large, making it difficult to achieve the direct digitization of the sensor output. Increasing the capacitance $C_{s}$ of the sensing probe itself is the main way to make the sensor meet the self-integration condition and improve the voltage division ratio. Therefore, this article equivalently increases the inherent capacitance $C_{s}$ of the sensor itself by paralleling ceramic capacitors in order to make the sensor operate in self-integration mode while increasing the sensor’s voltage division ratio.

**Figure 3:**
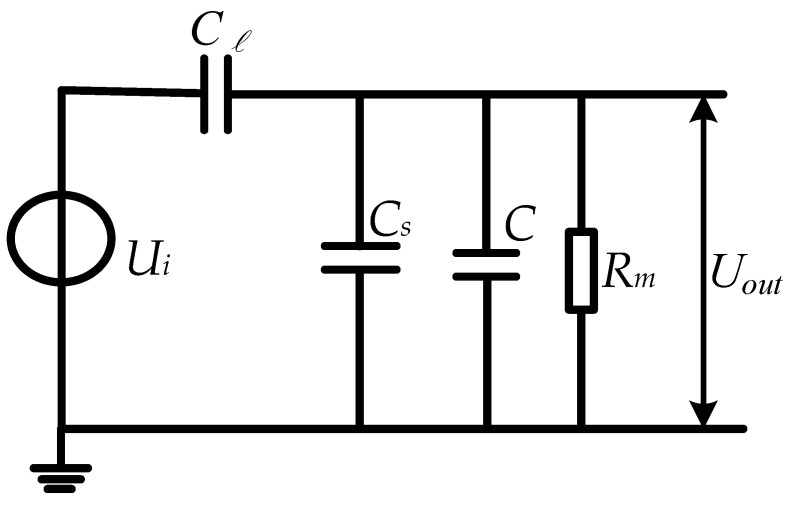
Equivalent schematic diagram after adding capacitors.

The equivalent schematic diagram after adding a parallel capacitor is shown in Figure 3. Following on from the sensor’s own capacitance $C_{s}$, the parallel capacitor C effectively increases the mutual capacitance value $\left(C_{s}+C\right)$ between the two sensing electrodes of the sensor. During this process, no changes were made to the sensor structure, induction electrode position, etc. Therefore, the parallel capacitance C between the two induction electrodes of the sensor only effectively increased the sensor’s own capacitance value from $C_{s}$ to $\left(C_{s}+C\right)$, and the coupling capacitance value $C_{l\;}$ between the sensor and the circuit to be tested was unchanged.

Through Laplace transformation, the transfer function of the sensor in this case is obtained as follows:

(5) $$H(s)=\frac{U_{o}(s)}{U_{i}(s)}=\frac{C_{l}R_{m}s}{(C_{l}+C_{s}+C)R_{m}s+1}$$

when $(C_{l}+C_{s}+C)R_{m}s+1>>1$, the transfer function can be expressed as follows:

(6) $$H\left(s\right)=\frac{U_{o}\left(s\right)}{U_{i}\left(s\right)}=\frac{C_{l}}{C_{l}+C_{s}+C}$$

At this point, the voltage division ratio $k_{2}$ of the sensor is as follows:

(7) $$k_{2}=\frac{U_{i}}{U_{o}}=\frac{C_{l}+C_{s}+C}{C_{l}}$$

In Equation (5), capacitor C is the lumped parameter capacitor connected in parallel to the back end of the sensor. Its capacitance value can be selected from nF level or even uF level, meaning that only Rm is MΩ to meet the self-integration condition $(C_{l}+C_{s}+C)R_{m}s+1>>1$, effectively improving the low-frequency performance of the sensor. Capacitors $C\gg C_{l},\;C_{s}$; therefore, the sensor voltage division ratio $k_{2}\gg k_{1}$ after the parallel capacitors significantly improve the sensor voltage division ratio, achieving the direct digitization of output voltage and the measurement of higher voltage levels.

From the above analysis, it can be inferred that the sensor can be equivalent to a high pass filter without parallel sampling capacitors at the back end of the sensor. The low-frequency performance of the sensor is determined by the turning frequency $\omega_{h1}$:

(8) $$\omega_{h1}=\frac{1}{(C_{l}+C_{s})R_{m}}$$

when the capacitance C is connected in parallel between the two induction electrodes of the sensor, the level of capacitance C is generally nF~uF, which is much greater than the sensor’s own capacitance $C_{s}$ and coupling capacitance $C_{l}$. At this time, the turning frequency $\omega_{h2}$ is as follows:

(9) $$\omega_{h2}=\frac{1}{(C_{l}+C_{s}+C)R_{m}}$$

**Figure 4:**
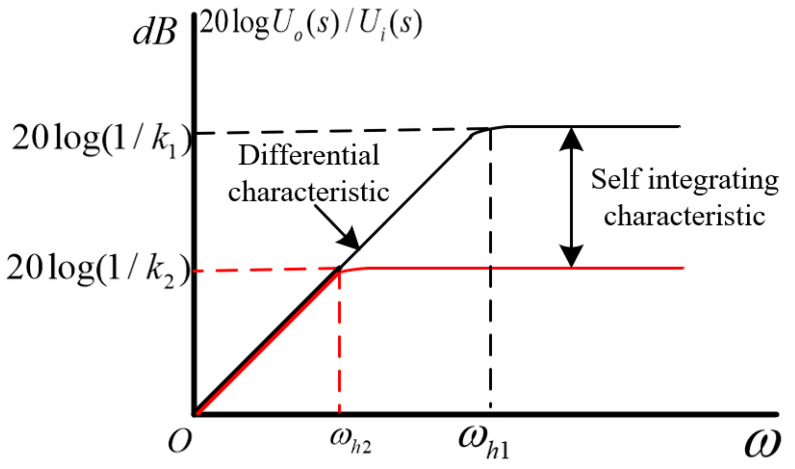
Amplitude–frequency response curves before and after shunt capacitor C.

Therefore, $\omega_{h2}<<\omega_{h1}$ improves the low-frequency performance of the sensor and effectively widens the sensor bandwidth. The amplitude frequency response curve of the sensor before and after the parallel capacitor C is represented in Figure 4. In the figure, black represents the absence of parallel capacitor C, while red represents the parallel capacitor C.

By paralleling a lumped capacitor behind the voltage sensor, the sensor operates in self-integration mode while increasing the sensor’s voltage division ratio, making the sensor output voltage digital and suitable for high-voltage measurement scenarios. However, this method becomes viable upon selecting capacitor C, the voltage division ratio of which, despite the fact that the method meets the self-integration working mode, is also determined by the selection of capacitance value. Sensors cannot measure multiple voltage levels at the same time (e.g., 400 V voltage level and 10 kV voltage level). In order to solve this problem, this paper introduces a SPDT switch to change the size of the parallel capacitor to achieve the switching of different sensor voltage division ratios.

**Figure 5:**
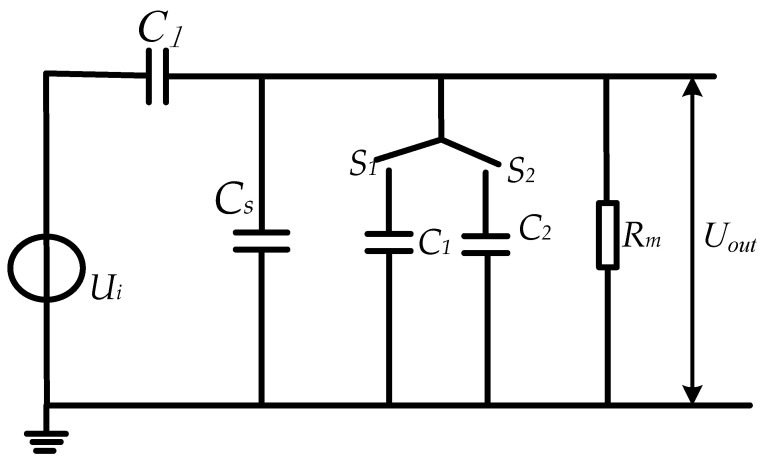
Equivalent circuit diagram of adjustable voltage division ratio.

As shown in Figure 5, by designing a controllable switch at the back end of the sensor, different parallel capacitance values are selected for different measurement scenarios. The switched capacitor needs to meet the following requirements: (1) meet the self-integration conditions of the sensor and improve the low-frequency characteristics of the sensor; (2) meet the voltage division ratio at the corresponding voltage level to ensure that its output voltage meets the collection range.

## 3. Voltage Sensor System Design

### 3.1. Voltage Sensor Structure Design

**Figure 6:**
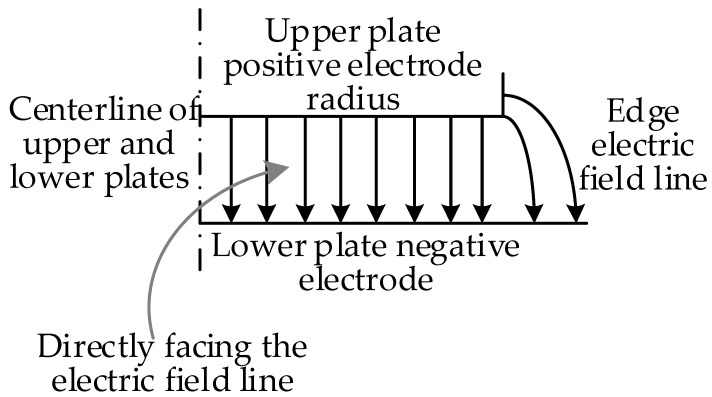
Distribution of the electric field lines of the sensor pole plate.

In an ideal situation, the electric field of traditional symmetrical structure sensors is uniformly distributed, but in reality, the electric field distribution in the middle part of the sensor plate is uniform, and the electric field lines at its edges are curved and divergent. This phenomenon of divergent electric field at the edges is called edge effect. Due to the edge effect of the symmetric structure sensor plate, there is an additional capacitance at the edge of the sensor plate, which means that the actual capacitance value of the sensor should be greater than the theoretically calculated capacitance value. The distribution of electric field lines on the electrode plate of a symmetrical structure sensor is shown in Figure 6.

According to the Gauss’s law of the electric field, the electric flux passing through any closed surface S is equal to the electric quantity surrounded by the closed surface divided by $\varepsilon_{o}$, independent of any charges other than S, which is

(10) $${∯}_{S}E\cdot dS=\frac{1}{\varepsilon_{o}}\underset{\;s}{\Sigma }q_{i}$$

From Equation (10), it can be seen that the total number of electric field lines emitted by the point charge q is $q/\varepsilon_{o}$. No matter how large a sphere is used to surround it, all electric field lines will pass through the sphere, without any omissions. This also explains why the actual value of the sensor plate is greater than the theoretically calculated capacitance value; i.e., the sensor plate electric field effect is the result of the combined action of the positive electric field effect and the edge electric field effect.

If the sensor plate with a symmetrical structure using air as the medium is analyzed, let the electric quantity |Q| carried by the upper and lower plates be the following:

(11) $$\left|Q\right|=\left|Q_{1}\right|+\left|Q_{2}\right|$$

In this equation, $Q_{1}$ is the electric charge of the area band directly opposite the upper and lower plates; $Q_{2}$ is the electrical charge of the upper and lower non aligned plates and the edge bands.

The calculation formula for symmetric structure sensors is as follows:

(12) $$C=\frac{\varepsilon_{o}S}{d}$$

The calculation formula for the potential difference U between the sensor plates is as follows:

(13) U=∫E·dl

Combining Equations (11) and (12) yields the following:

(14) $$C=\frac{Q}{U}=\frac{Q_{1}}{U}+\frac{Q_{2}}{U}$$

If $\sigma_{1}$ is the charge surface density of the area directly opposite the upper and lower plates, then

(15) $$\frac{Q_{1}}{U}=\frac{\sigma_{1}S}{\int E\cdot d\overset{\sim }{L}}=\frac{\sigma_{1}S}{\int_{A}^{B}\frac{\sigma_{1}}{\varepsilon_{o}}dr}=\frac{\varepsilon_{o}S}{d}$$

In Equations (14) and (15), U represents the voltage between the upper and lower plates of the sensor, S represents the positive area, and d represents the distance between the upper and lower poles. Equation (15) represents the capacitance of the sensor plate without considering edge effects, which clearly states that

When $\frac{Q_{1}}{U}>>\frac{Q_{2}}{U}$, there is

(16) $$C\approx \frac{Q_{1}}{U}=\frac{\varepsilon_{o}S}{d}$$

When considering edge effects, its capacitance should be

(17) $$C=\frac{Q}{U}=\frac{Q_{1}}{U}+\frac{Q_{2}}{U}=\frac{\varepsilon_{o}S}{d}+\frac{Q_{2}}{U}$$

If $\frac{Q_{1}}{U}$ and $\frac{Q_{2}}{U}$ are approximately equal, Equation (16) cannot accurately express the capacitance value and Equation (17) must be used. From this, it can be seen that when $Q_{1}$ is constant, the smaller $Q_{2}$, the closer the capacitance value calculated by Equation (16) is to the actual capacitance value.

**Figure 7:**
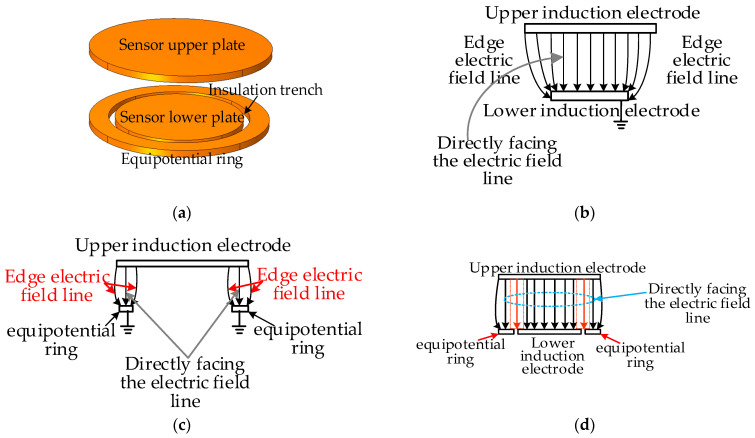
Schematic diagram of sensor structure: (**a**) schematic diagram of sensor structure with equipotential ring; (**b**) electric field distribution diagram of the upper and lower induction electrode sections; (**c**) electric field distribution diagram of the upper induction electrode and equipotential ring section; (**d**) electric field distribution diagram of sensor section with equipotential ring.

To reduce the edge effect caused by the sensor sensing plate, the actual value between the sensor plates is greater than the theoretically calculated capacitance value. For this article, we designed the sensing electrode structure by adding an equipotential ring structure at the position of the lower electrode plate of the sensor, as shown in Figure 7a. The radius of the upper plate of the sensor is the same as the outer radius of the equipotential ring. Adding an equipotential ring can ensure that the electric field between the upper and lower plates of the same area as the lower plate of the sensor is a uniform electric field. The specific implementation principle is as follows: Figure 7b shows the electric field distribution diagram when there are only upper and lower induction plates. The grounding of the lower induction electrode can indicate the presence of edge electric fields at the two edges of the lower plate; Figure 7c shows the distribution of the electric field when there are only the upper induction electrode plate and the equipotential ring. The equipotential ring is grounded like the lower induction electrode, indicating that there is also an edge electric field at the two edges of the equipotential ring. However, the deviation direction of the edge electric field near the lower plate of the equipotential ring is opposite to that in Figure 7b. In Figure 7d, the distorted electric field line at the edge position is represented by a dashed line. It can be seen that the deviation direction of the edge electric field at the equipotential ring and the lower induction electrode is opposite, meaning that the direction of the combined electric field strength here will be approximately the same as the direction of the electrode directly opposite to the plate to weaken the edge effect of the sensing electrode. The actual capacitance value of the sensor is approximately equal to the theoretical calculated capacitance value.

**Figure 8:**
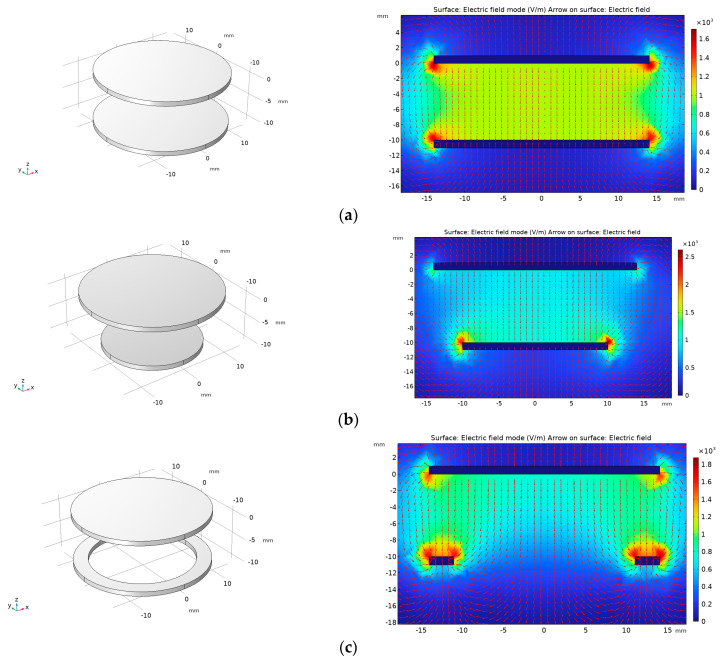

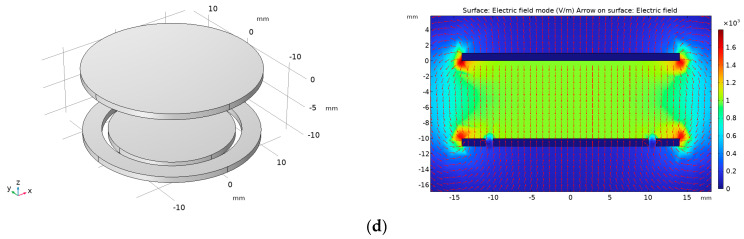
Finite element simulation model and simulation results: (**a**) traditional symmetrical electrodes with equal upper and lower induction electrode areas; (**b**) the cross-sectional electric field distribution map when the area of the upper induction electrode is greater than that of the lower induction electrode; (**c**) their cross-sectional electric field distribution map when the upper induction electrode and equipotential ring are added; (**d**) the distribution of electric field at the position where the two induction electrodes of the sensor are facing each other after the addition of the equipotential ring.

Below, we describe how we used the COMSOL simulation software (COMSOL® 6.1) to simulate and visualize the edge effect of the electric field line at the edge of the traditional symmetrical electrode mentioned above, which is curved and divergent. At the same time, simulation verification is conducted based on the proposed use of equipotential ring structure to weaken the edge effect between sensor electrodes. During the simulation process, the thickness of the induction electrodes is set to 1 mm, the radius of the upper induction electrode is set to 14 mm, and a 10 V voltage is applied. The lower induction electrode and equipotential ring are grounded, with a distance of 10 mm between the upper and lower induction electrodes, and the intermediate insulation medium is air. Figure 8 shows the COMSOL simulation model and its corresponding cross-sectional electric field distribution diagram. In the figure, (a) shows the traditional symmetrical electrode, with the same upper and lower induction electrode radii of 14 mm. It can be seen that the electric field lines at the edge positions are curved and diverging, and there is a significant edge effect; (b) the radius of the induction electrode in the figure is 14 mm, and the radius of the lower induction electrode is 10 mm. It can be seen that there is an electric field line that bends inward and diverges towards the lower induction electrode at the edge position of the lower induction electrode; (c) the figure retains the upper induction electrode, removes the lower induction electrode, and adds an equipotential ring structure (with an inner diameter of 11 mm and an outer diameter of 14 mm). It can be seen that the electric field line at the edge of the equipotential ring bends inward; (d) the figure shows the sensor model with an equipotential ring proposed in this article (the upper induction electrode radius is 14 mm, the lower induction electrode radius is 10 mm, the inner diameter of the equipotential ring is 11 mm, the outer diameter is 14 mm, and the distance between the upper and lower parts is 10 mm). From the simulation results, it can be seen that adding an equipotential ring structure can eliminate the edge effect of the electrode plate and ensure that the electric field at the position where the upper and lower induction electrodes of the sensor are directly opposite is a uniform electric field.

The following paragraphs further explore the relationship between the width of the insulation trench and the edge effect after introducing an equipotential ring, providing a basis for subsequent sensor structure design. To make the simulation data quantifiable, the method adopted involves directly simulating and obtaining the longitudinal average electric field mode size at the edge position, comparing it with the uniform electric field mode size, and calculating the distortion error γ% between them. The calculation formula is as follows:

(18) $$\gamma \%=\frac{E^{\prime }-E}{E}\times 100\%$$

In this equation, $E^{\prime }$ is the average longitudinal electric field mode at the edge of the induction electrode plate, and E is the theoretical electric field mode between the two induction electrodes, which is 1000 V/m (during all simulation processes, the distance between the upper and lower induction electrodes is kept at 10 mm, the upper induction electrode is charged with 10 V, and the lower induction electrode and equipotential ring are grounded).

**Figure 9:**
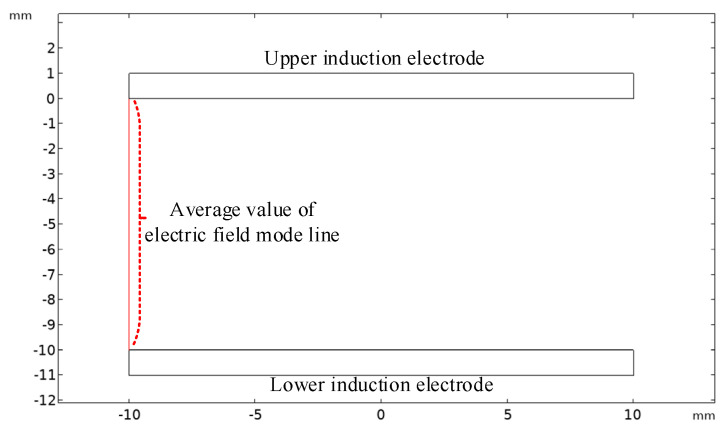
Average value of electric field mode line at the edge when the area of the upper and lower induction electrodes is equal.

As shown in Figure 9, when the radii of the upper and lower induction electrodes are both 10 mm, the average value of the electric field mode line at the edge of the sensor plate is 1041.5 V/m. By substituting Equation (18), the distortion error γ% can be calculated to be 4.15%.

**Figure 10:**
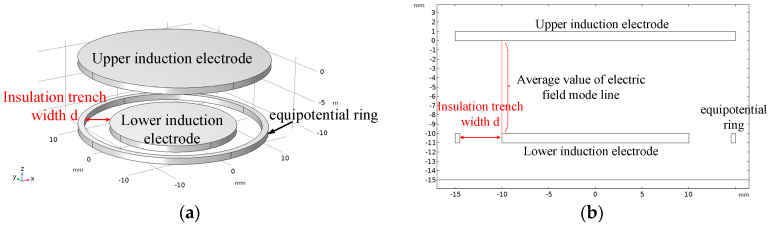
Effect of insulation trench width on edge electric field after adding equipotential rings: (**a**) simulation model; (**b**) section diagram of simulation model.

As shown in Figure 10, parameterized scanning of the electric field distortion error under different insulation trench widths d is performed to determine the optimal structure. During the simulation process, in which the upper induction electrode radius of 15 mm and the lower induction electrode of 10 mm were maintained, the distortion error γ% values under different insulation trench widths d were obtained (Table 1).

**Figure 11:**
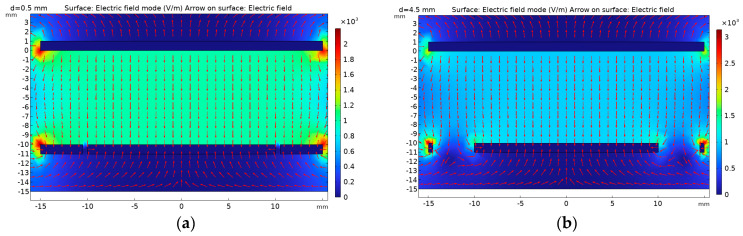
Tangential electric field distribution under different insulation trench widths: (**a**) the cross-sectional electric field distribution map when the width of the insulation trench is d = 0.5 mm; (**b**) the cross-sectional electric field distribution diagram when the width of the insulation trench d = 4.5 mm.

From Table 1, it can be seen that the larger the width d of the insulation trench, the greater the distortion error γ% of the edge electric field. This means that in the actual design process, the width of the insulation trench should be minimized as much as possible to ensure that the electric field is uniformly distributed at the edge positions of the upper and lower induction electrodes. As shown in Figure 11, the tangential electric field distribution at the minimum insulation trench width of 0.5 mm and the maximum insulation trench width of 4.5 mm clearly shows that the electric field distortion at the edge positions of the upper and lower induction electrodes is more severe when d = 4.5.

**Figure 12:**
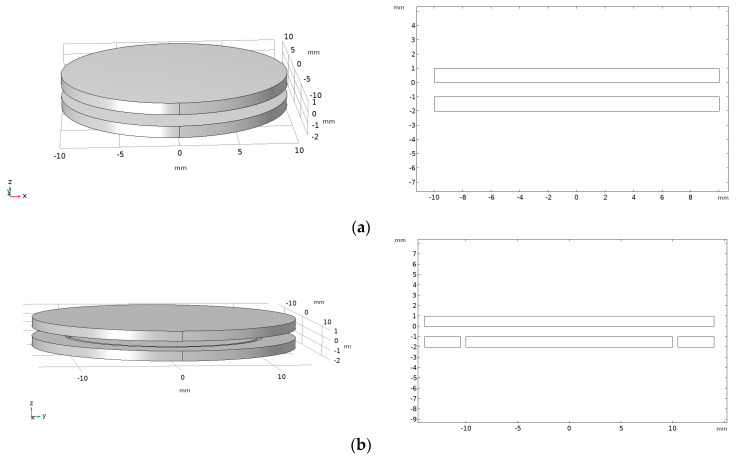
Simulation model and section diagram for extracting lumped capacitance: (**a**) simulation model and section diagram without incorporating equipotential loops; (**b**) simulation model and section diagram with equipotential ring added.

In order to verify the proposed method of adding an equipotential ring at the sensing electrode below the sensor to improve the edge effect of the sensor and ensure that the actual capacitance value of the sensor is approximately equal to the theoretical calculation value, COMSOL simulation was used to calculate the capacitance value of the sensor without the equipotential ring and the actual capacitance value after the equipotential ring is added, and the deviation between the capacitance value and the theoretical calculation value was calculated. The simulation model is shown in Figure 12, where Figure 12a shows a traditional symmetrical structure without an equipotential ring structure. The thickness of the upper and lower induction electrodes is 1 mm, the radius is 10 mm, and the default air filling spacing in the middle is 1 mm; Figure 12b shows the sensor structure with an equipotential ring structure, where the upper sensing electrode is 12 mm, the lower sensing electrode is 10 mm, and the thickness of the electrode plates is 1 mm. The default air filling spacing between the electrode plates is 1 mm, ensuring that the area, insulation medium, and thickness of the two electrode plates are exactly the same. Add an equipotential ring with an outer diameter of 12 mm and an inner diameter of 10.5 mm.

The theoretical calculated capacitance value is as follows:

(19) $$C=\frac{\varepsilon_{o}S}{d}=\;2.7816\;\mathrm{pF}$$

Here, $\varepsilon_{o}$ is the dielectric constant in vacuum, *S* is the face to face area of the sensor, which is a circular surface with a radius of 10 mm, and *d* is the distance between the two induction electrodes of the sensor, which is taken as 1 mm.

The percentage deviation between the capacitance value of the sensor before and after adding the equipotential ring and the theoretical calculated capacitance value is shown in Table 2.

From the above simulation results, it can be seen that adding an equipotential ring structure to the sensor’s structure can weaken the edge effect at the position of the sensing electrode, making the capacitance value of the sensor closer to the theoretical calculated true value.

**Figure 13:**
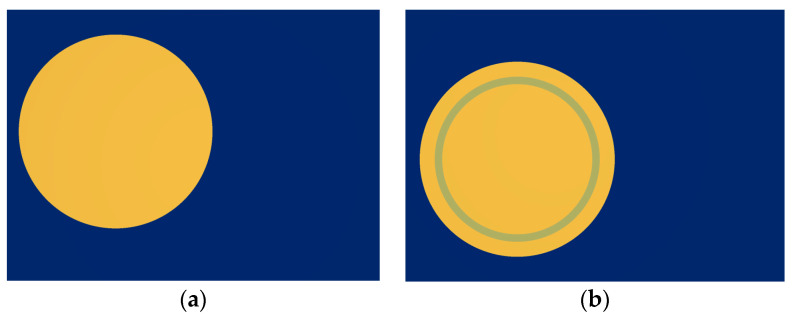
PCB diagram of voltage sensor prototype: (**a**) upper induction electrode on the top layer; (**b**) lower induction electrode and equipotential ring on the bottom layer.

Finally, to ensure the accuracy and standardization of the sensor’s own parameters, a non-contact voltage sensor was made in the form of a printed circuit board (PCB), as shown in Figure 13. In the figure, (a) shows the upper sensing electrode of the sensor, while (b) shows the lower sensing electrode and equipotential ring of the sensor.

### 3.2. Design of Back-End Signal Processing Circuit

Due to the need to parallel capacitors at the back end of the sensor to operate in self-integration mode, the sensor has broadband characteristics. At the same time, in order to make the sensor suitable for different voltage levels, the voltage division ratio of the sensor can be adjusted by switching the size of the parallel capacitor. Therefore, the capacitance connected in parallel at the back end of the sensor should have both (1) the ability to operate the sensor in self integrating mode and (2) the ability to meet the minimum voltage division under the corresponding voltage level so that the output voltage meets the collection conditions.

**Figure 14:**
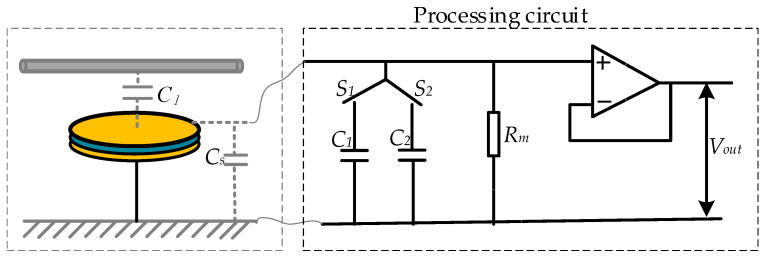
Circuit schematic analysis of sensor measurement system.

To ensure that the back-end acquisition does not affect the overall performance of the front-end sensor, a voltage follower is added to the circuit for input and output isolation processing. The model of the operational amplifier is OPA131UA produced by Texas Instruments Incorporated (TI) in the United States, with a bandwidth of 4 MHz, a maximum bias current of 50 pA, an input impedance of 10 GΩ, and a commonly used power supply voltage of ±5 V. The specific circuit schematic diagram is shown in Figure 14.

**Figure 15:**
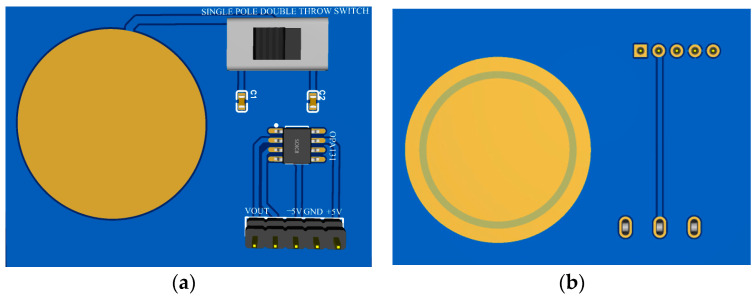
PCB diagram of sensor measurement system: (**a**) top layer of PCB; (**b**) bottom layer of PCB.

Finally, the sensor measurement system, as shown in Figure 15, integrates the sensor sensing electrode and back-end processing circuit on the same PCB board, effectively reducing the overall volume of the sensor to miniaturize the sensor.

## 4. Experimental Testing and Result Analysis

### 4.1. Low-Voltage (400 V) Performance Test

According to the above analysis, the size of the parallel capacitor and the value of the resistance $R_{m}$ in the back-end processing circuit of the voltage sensor need to meet the self-integration condition of $(C_{l}+C_{s}+C)R_{m}s+1>>1$. In the actual measurement process, the resistance $R_{m}$ value is 10 MΩ, which is used to calculate the value of the parallel lumped parameter capacitance. Due to $C>>C_{l},C_{s}$, the capacitance C only needs to meet $C>>\frac{1}{R_{m}s}$ to achieve sensor operation in self-integration mode. Take s=jω=j2πf as the minimum measurement frequency f=50 Hz and calculate $C>>3.1831\times 10^{-10}F$. Finally, select a chip ceramic capacitor with a nominal value of 1.2 nF. At this point, the minimum capacitance value is selected. If the output voltage is too large to meet the collection standard due to the small sensor voltage division ratio during the subsequent measurement process, a larger capacitance value can be selected to reduce the sensor voltage division ratio.

**Figure 16:**
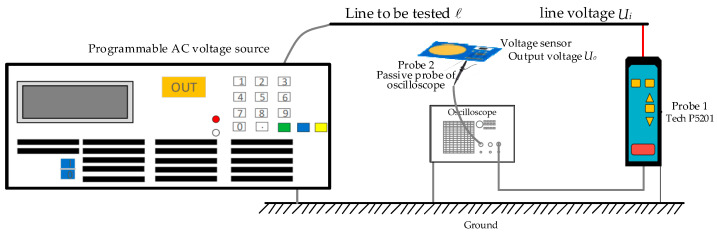
Schematic diagram of the low-voltage performance testing experiment used to test the sensors.

**Figure 17:**
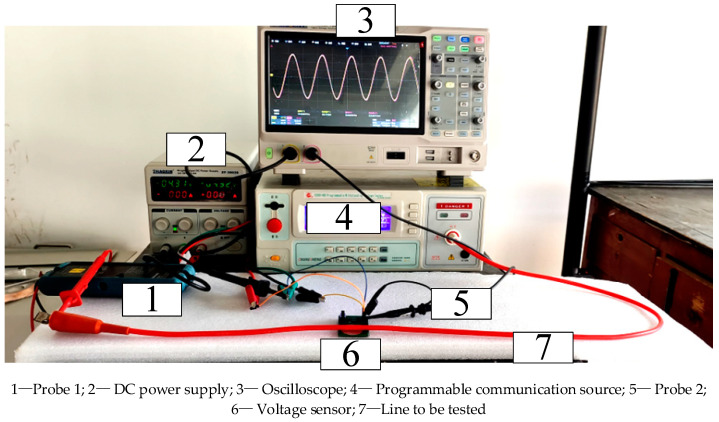
Experimental platform for the low-voltage performance testing of the sensors.

In order to study the testing performance of sensors in low-voltage scenarios, a low-voltage testing platform, a schematic diagram of which is shown in Figure 16, and a testing experimental platform, as shown in Figure 17, were constructed. In the figure, the DC voltage source provides ±5 V DC voltage to power the OPA131UA operational amplifier. The programmable AC voltage source is CS9914BX produced by Nanjing Changsheng Instrument Co., Ltd. in China, (output voltage range: 0.05–5.0 kV, frequency: 40–400 Hz) to output the voltage to be tested with a frequency of 50 Hz. The frequency was increased from 100 V to 400 V in steps of 20 V. The true value Ui of the line voltage to be tested was measured using the sensing probe 1 Tektronix P5201 produced by Tektronix Inc. in the United States (measurement voltage peak: 1400 V, bandwidth: 25 MHz). In the experiment, the voltage sensor was placed below the circuit to be tested, and the output voltage Uo of the sensor was measured using the SP2035A passive voltage probe model matched with the sensor probe 2 oscilloscope (with a measured voltage effective value of 300 V and a bandwidth of 350 MHz). Probe 1 and Probe 2 (shown in Figure 17) were connected to the SIGLENT SDS200X PLUS oscilloscope channel produced by SIGLENT Technologies Co., Ltd. in Shenzhen, China to obtain voltage values. The oscilloscope had a simulated bandwidth of 500 MHz and a real-time sampling rate of up to 2 GSa/s.

Additionally, we simultaneously measured the true value *U_i_* of the circuit voltage output from sensor probe 1 and the output voltage *U_o_* of sensor probe 2. The measurement results are shown in Table 3.

**Figure 18:**
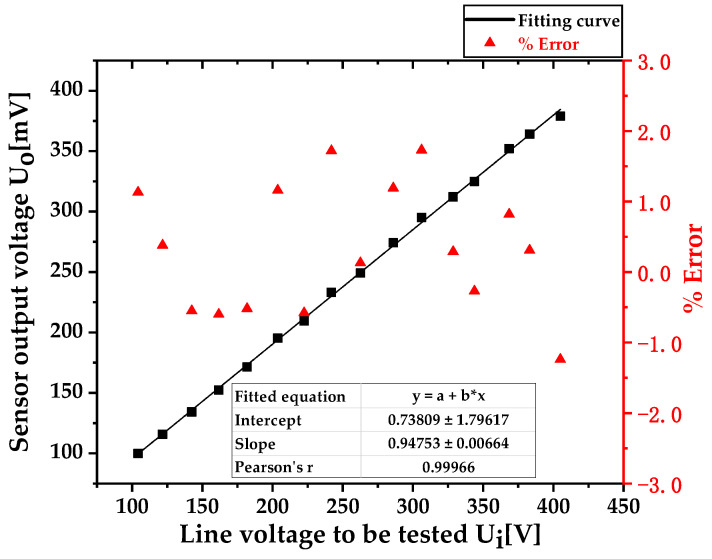
Sensor linearity and accuracy in low-voltage (400 V) scenarios.

Fit the actual voltage value $U_{i}$ of the tested line voltage in Table 3 with the sensor output voltage value $U_{o}$; the fitting results are shown in Figure 18. From the fitting results, the expression of the fitting curve can be obtained as y = 0.94753x + 0.73809, where x is the voltage value of the tested line (in V); y is the output voltage of the voltage sensor (in mV). The slope of the fitting curve is 0.94753, which represents the resolution of the sensor. The voltage value of the tested line changes by 1 V, and the output voltage of the sensor changes by 0.94753 mV. Therefore, the rated voltage ratio $K_{n}$ of the sensor under this working condition was calculated to be 1 V/0.94753 mV = 1055.376, and a Pearson’s correlation coefficient of 0.99966 indicated that the sensor had good linearity.

The formula for calculating the ratio error ε% of the sensor is as follows:

(20) $$\varepsilon \%=\frac{K_{n}U_{o}-U_{i}}{U_{i}}\times 100\%$$

In Equation (20), $K_{n}$ is the rated partial pressure ratio of the sensor obtained from the fitted curve, which, under this operating condition, is 1055.376; $U_{o}$ is the output voltage value of the voltage sensor, and $U_{i}$ is the true voltage value of the tested circuit measured by the reference probe.

In Figure 18, the horizontal axis represents the output voltage value of the reference probe 1 Tektronix P5201, which is the actual voltage value $U_{i}$ of the tested circuit. The left axis represents the sensor output voltage value $U_{o}$ measured by probe 2, and the right axis represents the calculated ratio error ε%. From the figure, it can be seen that the sensor has good linearity in the low voltage 400 V measurement scenario and that the measurement error is within ±2.0%.

### 4.2. High-Voltage (10 kV) Performance Test

The above experiment involved testing the low-voltage performance of the voltage sensor in 400 V application scenarios, and its performance is good in low-voltage scenarios, as evidenced by the sensor exhibiting high testing accuracy. In order to verify the proposed method of switching the capacitance of the back-end parallel capacitor to achieve the switching of the sensor’s voltage division ratio, the voltage sensor can be applied to different voltage measurement scenarios and has a certain degree of adaptability. In order to apply the sensor to a 10 kV voltage measurement scenario, the back-end parallel capacitance value is selected as 10 nF to increase the sensor’s voltage division ratio. Based on the above calculation, the capacitance value needs to be much greater than the voltage sensor to operate in self-integration mode. Therefore, in high-voltage scenarios, paralleling a 10 nF capacitor ensures that the sensor operates in self-integration mode while effectively increasing the voltage division ratio of the sensor.

**Figure 19:**
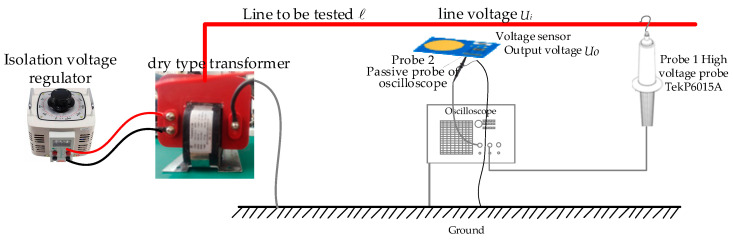
Schematic diagram of the high-voltage performance test.

**Figure 20:**
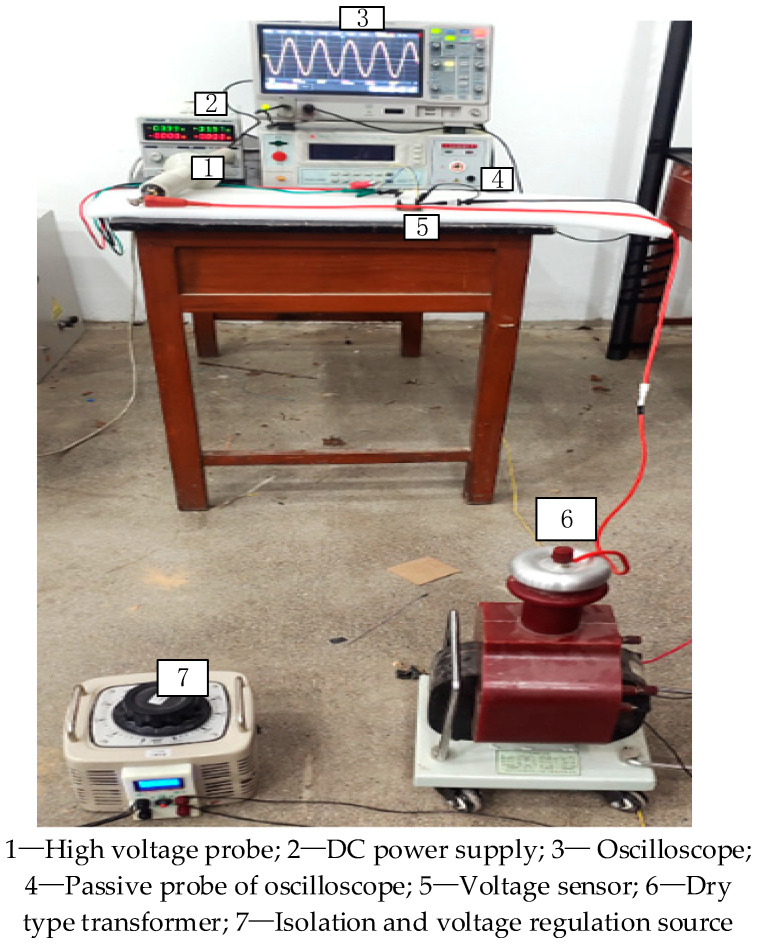
Experimental platform for high-voltage performance testing.

A schematic diagram of the high-voltage performance test and the experimental platform of the built high-voltage test are shown in Figure 19 and Figure 20. The difference between this experimental platform and the low-voltage testing platform is that, in high-voltage measurement scenarios, isolation voltage regulators (adjustable range: 0–500 V) and dry-type transformers GTB-5/50 (conversion ratio: 1:250 V) are used to boost voltage to generate up to 10 kV of the tested voltage value. The output voltage frequency is 50 Hz, and the voltage is raised from 500 V to 10 kV in steps of 500 V. At the same time, probe 1 TekP6015A produced by Tektronix Inc. in the United States (measuring voltage up to 20 kV, bandwidth DC-75 MHz) is used to monitor the measured voltage to determine the true value of the measured line voltage. We simultaneously measured the output voltage of the high-voltage probe and the output voltage of the voltage sensor, and the measurement results are shown in Table 4.

**Figure 21:**
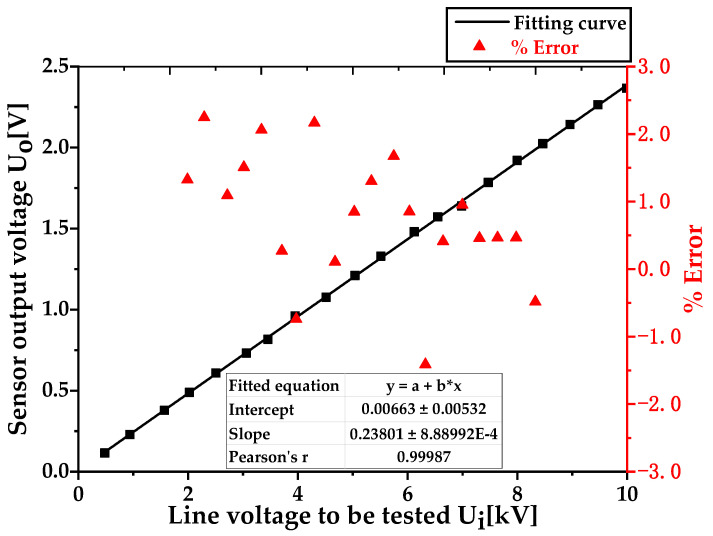
Sensor linearity and accuracy in high-voltage (10 kV) scenarios.

We fit the actual voltage value $U_{i}$ of the tested line voltage in Table 4 with the sensor output voltage value $U_{o}$, and the fitting results are shown in Figure 21. From the fitting results, the expression of the fitting curve can be obtained as y = 0.23801x + 0.00663, where x is the voltage value of the tested line (in kV); y is the output voltage of the voltage sensor (in V). The slope of the fitting curve is 0.23801, which represents the resolution of the sensor. The voltage value of the tested line changes by 1 kV, and the output voltage of the sensor changes by 0.23801 V. Therefore, the rated voltage ratio $K_{n}$ of the sensor under this working condition is calculated as 1 kV/0.23801 V = 4201.504. We substituted Equation (20) to calculate the ratio error ε%, and a Pearson’s correlation coefficient of 0.99987 indicated that the sensor has good linearity.

In Figure 21, the horizontal axis represents the output voltage of the isolation voltage regulator measured by TekP6015A of probe 1 and boosted by a dry transformer, which is the actual voltage value *U_i_* of the tested circuit. The left axis represents the sensor output voltage value *U_o_* measured by probe 2, and the right axis represents the ratio error ε%. From the figure, it can be seen that the sensor has good linearity in the low voltage 10 kV measurement scenario and that the measurement error is within ±3.0%.

In summary, the non-contact voltage measurement method based on the principle of electric field coupling proposed in this article enables non-contact voltage sensors to have certain adaptability. By paralleling a ceramic capacitor on the back-end of the induction electrode, the sensor’s own capacitance is effectively increased, significantly improving the sensor’s voltage division ratio and improving the low-frequency characteristics of the sensor. The sensor operates in self-integration mode at a frequency of 50 Hz (or even lower), effectively expanding the sensor frequency band. In addition, by adding a single pole double throw switch to control the size of the back-end parallel capacitor and change the sensor’s voltage division ratio, the sensor has a certain level of adaptability, meaning that it can meet voltage measurements in different measurement scenarios. Our tests showed that the sensor has good linearity and that the measurement error is within ±3% in both low-voltage (400 V) measurement scenarios and high-voltage (10 kV) measurement scenarios.

## 5. Conclusions

This article has proposed a non-contact voltage measurement method based on the principle of electric field coupling that can significantly improve the voltage sensor voltage division ratio, broaden the sensor bandwidth, and adapt to different measurement scenarios with variable sensor voltage division ratios. This measurement method is expected to be widely used in the field of non-contact voltage measurement, and the main points of this work can be summarized as follows:

(1) Based on the principle of electric field coupling, starting from the coupling capacitance between the sensor and the circuit to be tested and the self capacitance between the sensor plates, a voltage measurement model that is equivalent to the traditional capacitive voltage sharing principle was constructed.

(2) Based on the equivalent circuit model, analyze the transmission relationship of the sensors and the influence of their own capacitance and coupling capacitance on their output voltage. Due to factors such as the volume of the sensor itself and the dielectric constant of the insulation medium, the capacitance value of the sensor itself is only at the (pF) level, resulting in poor low-frequency performance and imbuing the sensing system with a small voltage division ratio, making it difficult to meet the application requirements in high-voltage scenarios.

(3) Through theoretical analysis, the edge effect at the edge of traditional symmetric induction electrodes can cause the actual capacitance value of the sensor to be greater than the theoretical capacitance value. Therefore, this article proposed an equipotential ring structure to eliminate edge effects and ensure that the electric field at the position where the two induction electrodes are facing each other is a uniform electric field. Finally, the results of this study were verified through COMSOL finite element simulation.

(4) To solve the problem of the small capacitance (pF level) of the sensor itself, as pointed out in (2), this paper proposed the notion of parallelly adding a ceramic capacitor with two sensing electrodes to increase the sensor’s capacitance value in order to improve the low-frequency performance of the sensor and significantly increase the sensor’s voltage division ratio.

(5) We also introduced a single pole double throw switch to change the size of the parallel ceramic capacitor, change the voltage division ratio of the sensor, and enable the sensor to be applied in different measurement scenarios (low-voltage and high-voltage scenarios) with certain adaptive ability.

(6) We utilized printed circuit boards (PCBs) to make sensor prototypes and build response testing platforms to test their performance and verify the feasibility of the method proposed in this paper.

On the basis of realizing non-contact and low-cost voltage sensors, this article solves the two major problems of poor low-frequency performance and low voltage division ratios caused by the small capacitance of the sensor itself. At the same time, by switching different capacitors, the sensor has a certain degree of adaptive ability. However, further research on certain aspects is still needed (e.g., considering the wireless signal transmission module and integrating it with sensors for research. In addition, when the measurement scenario is not a single line with multiphase power lines, shielding or multiphase decoupling algorithm research needs to be conducted.

## Figures and Tables

**Table 1:** Distortion error γ% values under different insulation trench widths d.

Insulation Trench Width d [mm]	Average Value of Electric Field Mode Line	Distortion Error γ%
0.5	1000.1	0.01
1	1000.2	0.02
1.5	1000.4	0.04
2	1002.8	0.28
2.5	1004.1	0.41
3	1003.5	0.35
3.5	1005.8	0.58
4	1017.5	1.75
4.5	1018.6	1.86

**Table 2:** Capacitance values before and after the addition of equipotential rings and their deviation from theoretical calculation values.

Sensor Structure Type	Capacitance Value (pF)	Percentage Deviation from Theoretical Value (%)
No equipotential ring structure	3.3270	19.8%
Adding an equipotential ring	2.8684	3.12%

**Table 3:** Input and output voltage and ratio error calculation results under a low-voltage (400 V) measurement scenario.

Line Voltage to Be Tested *U_i_* (V)	Sensor Output Voltage *U_o_* (mV)	Voltage Division Ratio	Ratio Errorε%
104.21	99.86	1044	1.13
121.69	115.74	1051	0.38
142.43	134.21	1061	−0.55
161.65	152.25	1062	−0.60
181.81	171.37	1061	−0.52
203.70	195.26	1043	1.16
222.45	209.56	1061	−0.58
241.87	233.12	1038	1.72
262.56	249.11	1054	0.13
285.97	274.19	1043	1.19
306.13	295.07	1037	1.73
328.55	312.21	1052	0.29
343.76	324.85	1058	−0.27
368.51	352.05	1047	0.82
383.09	364.12	1052	0.31
405.01	379.01	1069	−1.24

**Table 4:** Calculation results regarding input and output voltage and ratio error in the high-voltage (10 kV) measurement scenario.

Line Voltage to Be Tested *U_i_* (kV)	Sensor Output Voltage *U_o_* (V)	Voltage Division Ratio	Ratio Errorε%
0.481	0.116	4147	1.33
0.941	0.229	4109	2.25
1.571	0.378	4156	1.09
2.024	0.489	4139	1.51
2.507	0.609	4117	2.06
3.063	0.731	4190	0.27
3.454	0.816	4233	−0.74
3.952	0.961	4112	2.17
4.516	1.076	4197	0.11
5.041	1.210	4166	0.85
5.512	1.329	4147	1.30
6.120	1.481	4132	1.67
6.549	1.572	4166	0.85
6.985	1.639	4262	−1.41
7.469	1.785	4184	0.41
7.995	1.921	4162	0.95
8.465	2.024	4182	0.46
8.958	2.142	4182	0.46
9.468	2.264	4182	0.47
9.989	2.366	4222	−0.48

## Data Availability

The data used to support the findings of this study are included within the article.
